# Risk of Hepatocellular Carcinoma or Hepatic Decompensation Associated With Steatotic Liver Disease in People With and Without HIV

**DOI:** 10.1093/ofid/ofag617

**Published:** 2026-10-07

**Authors:** Jessie Torgersen, Craig W Newcomb, Melissa Skanderson, Farah Kidwai-Khan, Dean M Carbonari, Janet P Tate, Catherine Mezzacappa, Emily J Cartwright, Debika Bhattacharya, Joseph K Lim, Jennifer C Price, Tamar H Taddei, Amy C Justice, Vincent Lo Re

**Affiliations:** Department of Medicine, Perelman School of Medicine, University of Pennsylvania, Philadelphia, Pennsylvania, USA; Department of Biostatistics, Epidemiology, and Informatics, Center for Clinical Epidemiology and Biostatistics, Center for Real-World Effectiveness and Safety of Therapeutics, Perelman School of Medicine, University of Pennsylvania, Philadelphia, Pennsylvania, USA; Department of Biostatistics, Epidemiology, and Informatics, Center for Clinical Epidemiology and Biostatistics, Center for Real-World Effectiveness and Safety of Therapeutics, Perelman School of Medicine, University of Pennsylvania, Philadelphia, Pennsylvania, USA; VA Connecticut Healthcare System, West Haven, Connecticut, USA; VA Connecticut Healthcare System, West Haven, Connecticut, USA; Department of Medicine, Yale School of Medicine, New Haven, Connecticut, USA; VA Connecticut Healthcare System, West Haven, Connecticut, USA; Department of Medicine, Yale School of Medicine, New Haven, Connecticut, USA; Center for Pharmacoepidemiology and Treatment Science, Institute for Health, Health Care Policy, and Aging Research, Rutgers University, New Brunswick, New Jersey, USA; VA Connecticut Healthcare System, West Haven, Connecticut, USA; Department of Medicine, Yale School of Medicine, New Haven, Connecticut, USA; VA Connecticut Healthcare System, West Haven, Connecticut, USA; Department of Medicine, Yale School of Medicine, New Haven, Connecticut, USA; Department of Medicine, Emory University School of Medicine, Atlanta, Georgia, USA; VA Greater Los Angeles Healthcare System and Department of Medicine, David Geffen School of Medicine at UCLA, Los Angeles, California, USA; VA Connecticut Healthcare System, West Haven, Connecticut, USA; Department of Medicine, Yale School of Medicine, New Haven, Connecticut, USA; Department of Medicine, University of California, San Francisco School of Medicine, San Francisco, California, USA; VA Connecticut Healthcare System, West Haven, Connecticut, USA; Department of Medicine, Yale School of Medicine, New Haven, Connecticut, USA; VA Connecticut Healthcare System, West Haven, Connecticut, USA; Department of Medicine, Yale School of Medicine, New Haven, Connecticut, USA; Division of Health Policy and Management, Yale School of Public Health, New Haven, Connecticut, USA; Center for Pharmacoepidemiology and Treatment Science, Institute for Health, Health Care Policy, and Aging Research, Rutgers University, New Brunswick, New Jersey, USA; Division of Infectious Diseases, Department of Medicine, Robert Wood Johnson Medical School, Rutgers University, New Brunswick, New Jersey, USA

**Keywords:** hepatic decompensation, hepatocellular carcinoma, natural language processing, steatotic liver disease

## Abstract

**Background:**

Steatotic liver disease (SLD) is highly prevalent in people with HIV (PWH), but factors influencing progression to hepatocellular carcinoma (HCC) and hepatic decompensation (HD) remain unclear. It is unknown if the risk of these outcomes associated with SLD differs by HIV status.

**Methods:**

Patients from the Veterans Aging Cohort Study-HIV (includes matched people without HIV [PWoH]) who underwent an ultrasound, computed tomography, or magnetic resonance imaging between 2001–2017 were included. Patients with diagnosed cirrhosis or chronic viral hepatitis were excluded. SLD was defined by a natural language processing algorithm; primary outcome of composite of HCC or HD was defined using International Classification of Diseases 9/10 codes and national cancer registry. Follow-up began on imaging study date and continued until incident HCC/HD, death, or 31 December 2024. Adjusted hazard ratios (aHRs) with 95% confidence intervals (CIs) of HCC/HD associated with SLD and HIV status were calculated.

**Results:**

Among 19 546 patients (mean age: 54 years; 96.6% male; 28.6% PWH), 13 617 (69.7%) had SLD identified by natural language processing. Rates of HCC/HD were lowest in PWoH and no SLD (18.0/10 000 person-years) and highest among PWH and SLD (46.8/10 000 person-years). When compared to PWoH without SLD, risk of HCC/HD was increased among PWH with SLD (aHR: 2.71; 95% CI: 2.10–3.49) and PWH without SLD (aHR: 1.62; 95% CI: 1.14–2.29), although no interaction between HIV and SLD was observed (*P* = .25).

**Conclusions:**

Among people without cirrhosis or chronic viral hepatitis, radiographic findings of SLD were associated with increased rates of HCC/HD, with the highest rate among PWH and SLD, independent of cardiometabolic factors, alcohol, and tobacco.

MAIN POINTS Radiology report of steatotic liver disease was associated with increased risk of hepatocellular carcinoma or hepatic decompensation in people without diagnosed cirrhosis or chronic viral hepatitis. The rates were highest in people with HIV and steatotic liver disease.

The global prevalence of steatotic liver disease (SLD) is estimated to be 38%, with a higher burden observed among people with HIV (PWH) [1, 2]. HIV may potentiate SLD through multiple mechanisms, including direct viral effects on hepatocytes, disruption of lipid metabolism, gut dysbiosis, and metabolic dysfunction associated with antiretroviral therapy [3–5]. In addition, chronic immune activation and systemic inflammation in PWH can exacerbate hepatic insulin resistance and oxidative stress, further promoting hepatic steatosis and progression to steatohepatitis [3]. Despite these biologically plausible mechanisms, the clinical consequences of SLD in PWH, and how they differ from people without HIV (PWoH), remain understudied.

While liver disease is a leading cause of non-AIDS mortality in PWH [6], it remains unknown if SLD confers a different risk of hepatocellular carcinoma or hepatic decompensation (HCC/HD) among PWH compared to PWoH. Small studies have demonstrated that PWH with SLD exhibit greater degrees of hepatic fibrosis and inflammation than PWoH, suggesting that HIV infection may accelerate SLD progression [7–9]. Immune activation and intrahepatic depletion of CD4+ T cells accompanying hepatic steatosis could further promote fibrogenesis and hepatocarcinogenesis, effects that may be amplified in the setting of HIV infection [10–12]. Taken together, HIV may enhance or modify the effect of SLD on the risk of HCC/HD, underscoring the need to understand these interactions and their clinical implications.

Efforts to study SLD in real-world settings have been constrained by challenges in accurately defining this common condition. Prior work relying on diagnostic codes has been limited by their poor sensitivity and specificity due to underdiagnosis of SLD [13, 14]. In attempts to overcome these limitations, investigators have employed surrogate markers—such as persistently elevated alanine aminotransferase (ALT) levels—to identify SLD [15, 16]. However, this approach is also vulnerable to misclassification, since aminotransferases may be elevated by a variety of conditions, not only SLD. Moreover, individuals with SLD may maintain normal aminotransferase levels across the disease spectrum [17]. Accurate identification of SLD in observational data is therefore critical to understanding the clinical consequences of this common chronic liver disease.

As clinical imaging has increasingly identified steatosis through intentional or opportunistic evaluation, applying natural language processing (NLP) algorithms to repositories of radiographic imaging reports offers the potential to accurately identify individuals with SLD [18, 19]. We previously developed and validated an NLP algorithm to identify hepatic steatosis key terms within clinical liver imaging reports within the Veterans Health Administration (VA), demonstrating excellent accuracy compared with expert clinician review [20]. When applied to patients within the Veterans Aging Cohort Study (VACS)-HIV cohort, the algorithm permitted determination of SLD status among PWH and PWoH. The classification of this large subcohort of individuals with clinical liver imaging by SLD status now allows for longitudinal evaluation to define the risk of liver complications associated with SLD and to evaluate whether results differ by HIV status.

This study evaluated associations between SLD and HCC/HD, defined by a composite of first diagnosis of HCC or HD, among people with clinical liver imaging. We evaluated the rates of HCC/HD by HIV and SLD status and determined if the impact of SLD on liver complications differed in PWH.

## METHODS

### Study Design and Data Source

We conducted a retrospective cohort study among patients in the VACS-HIV (2001–2024), an ongoing electronic health record (EHR)-based cohort of PWH and 1:2 age-, sex-, race/ethnicity-, and clinical site-matched PWoH in care within VA facilities across the United States [21]. Data available within the VA EHR include hospital and outpatient diagnoses (recorded using International Classification of Diseases [ICD] 9 and 10 codes), procedures (recorded using Current Procedural Terminology codes), imaging reports, laboratory results, and dispensed medications. Because some patients within the VA have Medicare coverage and may have health care encounters outside of the VA, we linked the VA EHR data with administrative data from the US Centers for Medicare and Medicaid Services to increase ascertainment of HCC and HD events. The study was approved by the institutional review boards of the VA Connecticut Healthcare System and Yale University and was conducted under a waiver of informed consent per 45 CFR §46.117(c).

### Study Patients

PWH and PWoH were eligible for inclusion in the analysis if their enrollment date in VACS-HIV was between 1 October 2001 and 30 September 2017. This date range was selected to capture more of the modern antiretroviral therapy era and avoid undue influence of older antiretrovirals (eg, dideoxynucleoside analogues: stavudine, didanosine, zidovudine, zalcitabine), which have been associated with SLD [22, 23]. To facilitate identification of SLD, patients were included if they completed ≥1 ultrasound (US), computed tomography (CT), or magnetic resonance imaging (MRI) that included the liver following enrollment [24, 25]. We defined the index date as the date of the first eligible clinical imaging study performed on or after 1 October 2001. If more than 1 clinical imaging study was completed on the index date, the first study completed on that date was selected for inclusion.

To facilitate ascertainment of baseline characteristics, analyses were restricted to patients in VA care for at least 2 years prior to completion of an US, CT, or MRI study. Patients were excluded from the analyses if, prior to the index date, they had: (1) prevalent diagnosis of HCC or HD, (2) ICD 9/10 code for cirrhosis, and (3) chronic viral hepatitis B or C as defined by laboratory values [26, 27]. Because cirrhosis is on the causal pathway in the development of HCC and HD, we excluded patients with diagnosed cirrhosis at baseline. In addition, we excluded patients with chronic viral hepatitis at baseline to isolate the effect of HIV on progression of SLD.

The 2 years prior to the index date represented the baseline period. Follow-up continued until an incident HCC or HD event, death, 6 months after the last VA diagnosis in any setting (suggesting loss to care), or 31 December 2024, whichever occurred first. Death date was determined from the VA Vital Status File.

### SLD Identification by NLP

The NLP algorithm for the identification of steatotic liver key terms was previously validated in the VA [20]. Briefly, clinical imaging reports for US, CT, or MRI inclusive of the liver and completed for any indication were identified through query of the EHR. The first relevant clinical imaging report between 2001–2017 was selected for analysis. The NLP algorithm was applied to the radiology reports to identify hepatic steatosis key terms, including “fatty” and “liver” or “hepat”; “steatosis”; “steatotic”; and “steatohepatitis” within the free text narrative of the radiologist's findings from the body or impression sections of the report. Reports with negation terms (ie, “no evidence of steatosis”) or no hepatic steatosis key terms identified by NLP were considered to have no SLD for the purposes of the analysis. Prior work demonstrated excellent accuracy of the NLP algorithm compared to expert clinician review with 100% sensitivity, 89.7% specificity, 88.5% positive predictive value, and 100% negative predictive value [20].

### Study Outcomes

The primary outcome was defined by a composite of first HCC or HD diagnosis. Validated HCC diagnoses were abstracted from the VA Cancer Registry database of patients with cancer who were diagnosed and/or treated in the VA system [28]. These diagnoses were previously validated in the VA based on clinician review of VA medical records, with positive predictive value of 96% (95% confidence interval [CI], 92–98%) [29]. Topography code C22.0 (liver) and morphology codes 8170–8180 (HCC) from the International Classification of Diseases for Oncology, Third Edition (ICD-O-3) [30], defined HCC diagnoses, consistent with Surveillance, Epidemiology, and End Results coding algorithms [29, 31]. To account for lags in reporting diagnoses in the cancer registry and minimize the likelihood of missing HCC events, we included validated ICD 9/10 diagnoses for HCC recorded in the VA or Medicare administrative claims, as we have previously done [32]. HD events were defined by one hospital discharge diagnosis or 2 ICD 9/10 outpatient diagnoses of ascites, spontaneous bacterial peritonitis, or variceal hemorrhage; diagnosis of hepatic failure was additionally included within the ICD-10 definition. These definitions were previously validated in the VA with positive predictive values of 91% (ICD-9; 95% CI, 77–98) and 81% (ICD-10; 95% CI, 70–90) [27, 33]. To capture HD events occurring outside of the VA, we additionally included ICD 9/10 diagnostic codes abstracted from Medicare administrative claims, as previously done [34]. Only the first eligible HCC or HD event was included in the analysis.

### Data Collection

We collected demographic and clinical variables from dates closest, but within 2 years prior, to the index date. Variables included age, sex, race, ethnicity, body mass index (BMI), HIV status, laboratory results (ALT, aspartate aminotransferase [AST], platelets), and select comorbidities, including diabetes mellitus, hypertension, hyperlipidemia, and alcohol use disorder (AUD), defined by 1 hospital or 2 ambulatory ICD 9/10 diagnoses [35]. Since statin use might reduce risk of liver disease [36], dispensed fills for any statin during the baseline period were abstracted to further delineate the impact of this treatment within the category of hyperlipidemia. Tobacco use (classified as current, former, or never) was determined from the VA's Health Factors Dataset [37].

Alcohol use was defined as a categorical variable including AUD diagnoses coupled with the most recent Alcohol Use Disorders Identification Test-Consumption (AUDIT-C) score [38] (Supplementary Methods). Since people with reported alcohol abstinence include a heterogeneous group of non-users, “sick quitters,” and underreported use, the lower risk alcohol use group served as the reference [39, 40].

For descriptive purposes, the Fibrosis-4 Index for Hepatic Fibrosis (FIB-4) was calculated using ALT, AST, and platelet count as follows: [age (years)×AST (U/L)]/[platelet count (10^9^/L)] × [ALT (U/L)]^1/2^ [41]. FIB-4 > 2.67 was considered suggestive of advanced hepatic fibrosis/cirrhosis [42]. This FIB-4 threshold has been shown to rule in advanced hepatic fibrosis/cirrhosis among both PWH and PWoH with specificity of ≥97% and negative predictive value of ≥83% [43, 44].

### Statistical Analysis

We evaluated differences in baseline characteristics by SLD status. The magnitude of differences in characteristics of patients with and without SLD was evaluated using standardized mean differences for continuous data and standardized difference in proportions for categorical variables. A meaningful difference between the groups was defined as a standardized difference of >0.10 [45].

We calculated unadjusted incidence rates of HCC/HD (events/10 000 person-years), stratified by HIV and SLD status. Cumulative incidence plots were created using the Aalen-Johansen estimator to illustrate the incidence of HCC/HD over follow-up by SLD and HIV status while accounting for competing risk of death; the plots were compared via Gray's tests [46]. We first used multivariable Cox regression to determine the hazard ratios (HRs) with 95% CIs of HCC/HD associated with SLD and evaluated for potential interaction between SLD and HIV status. We then used multivariable Cox regression to determine the HRs with 95% CIs of HCC/HD associated 4 groups classified by SLD and HIV status: (1) PWH and SLD, (2) PWH and no SLD, (3) PWoH and SLD, and (4) PWoH and no SLD for descriptive clarity. We then determined the HRs of HCC/HD for groups 1–3 compared to PWoH and no SLD. The adjusted model included baseline age, sex, race, ethnicity, and comorbidities known to be associated with SLD and HCC or HD (ie, obesity, diabetes, hypertension, hyperlipidemia/statin use, alcohol use, and smoking).

We performed 2 sensitivity analyses to evaluate the robustness of our findings. First, since mortality rates may be higher in people with SLD and death could occur prior to the development of liver complications, we repeated the primary analyses accounting for the competing risk of death with subdistribution hazards models [47]. Second, since cirrhosis is clinically silent and may be underdiagnosed, we repeated the analyses excluding patients with advanced fibrosis/cirrhosis by baseline FIB-4 > 2.67, a threshold used with SLD [48]. We further explored rates of HCC/HD by baseline FIB-4 category and the four SLD and HIV groups.

All statistical analyses were performed using SAS v9.4 (SAS Institute Inc.).

## RESULTS

We identified 87 562 people within VACS-HIV with at least 2 years of VA care between 2001 and 2017. During this period, 75 968 people underwent a radiology study within the VA, of whom 26 706 had a CT, MRI, or US inclusive of the liver as the first eligible study. After excluding people with prevalent HCC, HD, cirrhosis, or chronic viral hepatitis, 19 546 remained (5929 PWH and 13 617 PWoH) and were included in the analytic sample (Figure 1). Overall, there was a high prevalence of comorbidities including BMI ≥ 30 kg/m^2^ (43%), diabetes (22%), hypertension (57%), and current tobacco use (42%).

**Figure 1.**
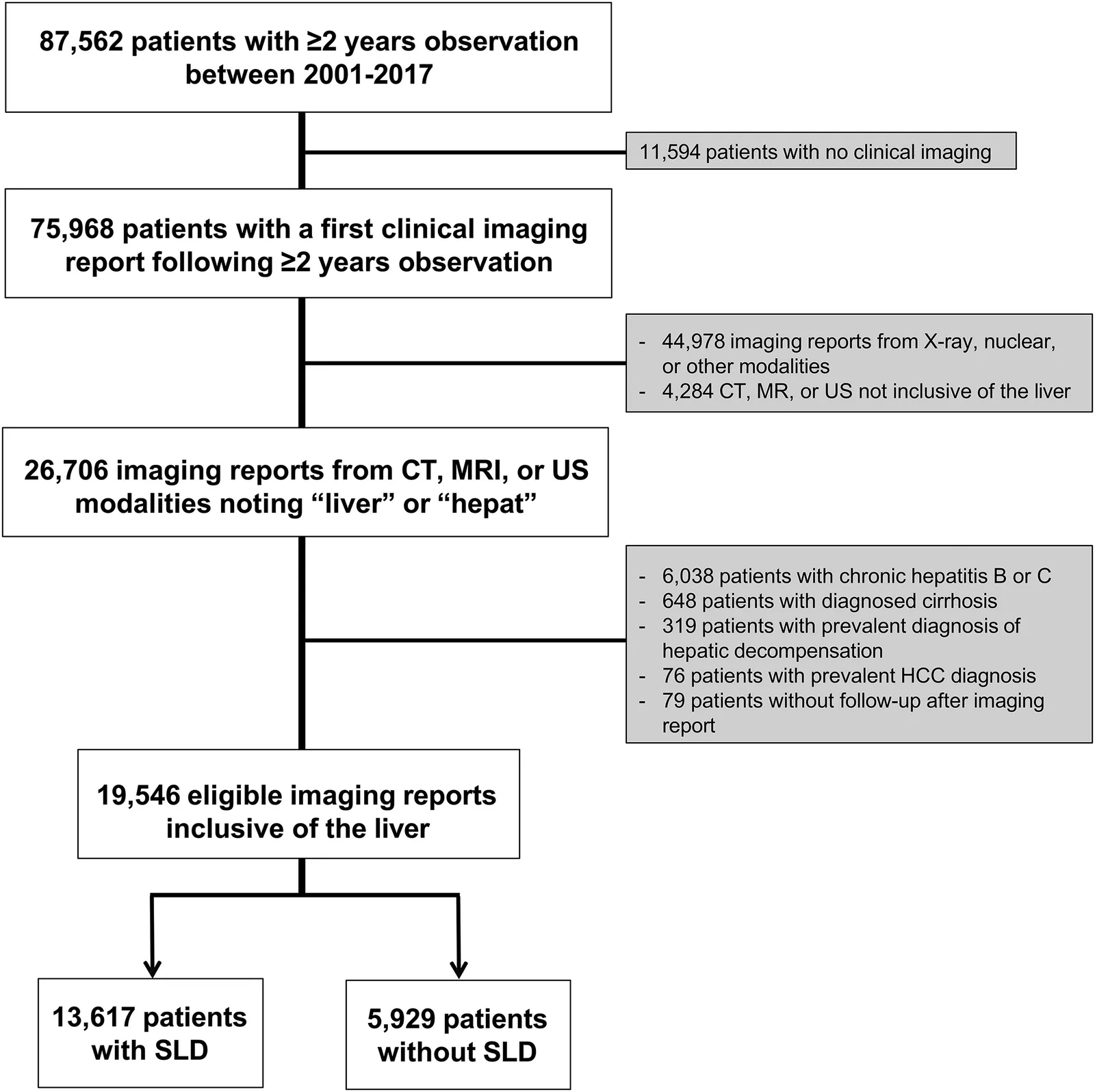
Selection of patients who met inclusion criteria for the study. Abbreviations: CT, computed tomography; HCC, hepatocellular carcinoma; MR, magnetic resonance; SLD, steatotic liver disease; US, ultrasound.

The NLP algorithm identified 69.7% with SLD and 30.3% without SLD within the sample. People with SLD were more commonly of Hispanic ethnicity, more likely to have a BMI in the obese range, diabetes, hypertension, high-risk alcohol use, and a FIB-4 score >2.67 (Table 1). Black race and HIV were more common among those without SLD.

**Table 1.** Characteristics of Patients With Clinical Liver Imaging in the VA Between 1 October 2001 and 30 September 2017 by Steatotic Liver Disease Status (n = 19 546)

Characteristics	No SLD (n = 5929)	SLD (n = 13 617)	Std. Diff.^a^
**Median (IQR) age, years**	55 (46–62)	53 (45–59)	0.14
**Male sex**	5738 (96.8%)	13 152 (96.6%)	0.01
**Race/ethnicity**			0.22
Non-Hispanic White	2288 (38.6%)	6219 (45.7%)	…
Non-Hispanic Black	2743 (46.3%)	4945 (36.3%)	…
Hispanic	345 (5.8%)	1232 (9.0%)	…
Other^b^	344 (5.8%)	854 (6.3%)	…
Unknown	209 (3.5%)	367 (2.7%)	…
**Body mass index, kg/m^2^**			0.36
<18.5	108 (1.8%)	151 (1.1%)	…
18.5–24.9	1641 (27.7%)	2392 (17.6%)	…
25.0–29.9	2137 (36.0%)	4297 (31.6%)	…
30.0–34.9	1226 (20.7%)	3692 (27.1%)	…
≥35.0	660 (11.1%)	2731 (20.1%)	…
Missing weight and/or height	157 (2.6%)	354 (2.6%)	…
**Tobacco use**			0.16
Never	1956 (33.0%)	4441 (32.6%)	…
Current	2598 (43.8%)	5705 (41.9%)	…
Former	1326 (22.4%)	3418 (25.1%)	…
Missing	49 (0.8%)	53 (0.4%)	…
**Diabetes mellitus**	1119 (18.9%)	3152 (23.1%)	0.11
**Hypertension**	3207 (54.1%)	7931 (58.2%)	0.08
**Hyperlipidemia/statin use**			0.05
Any statin use	2136 (36.0%)	5046 (37.1%)	…
Hyperlipidemia diagnosis, no statin use	445 (7.5%)	1169 (8.6%)	…
No hyperlipidemia diagnosis, no statin use	3348 (56.5%)	7402 (54.4%)	…
**Median (IQR) AUDIT-C score**	1.0 (0.0–2.0)	1.0 (0.0–2.0)	0.04
**Alcohol use category** ^c^			0.24
Abstinent without alcohol use disorder	1369 (23.1%)	2488 (18.3%)	…
Abstinent with alcohol use disorder	357 (6.0%)	613 (4.5%)	…
Lower risk	1220 (20.6%)	2183 (16.0%)	…
Moderate risk	246 (4.1%)	441 (3.2%)	…
High risk	881 (14.9%)	2492 (18.3%)	…
Missing AUDIT-C	1856 (31.3%)	5400 (39.7%)	…
**HIV infection**	1833 (30.9%)	3752 (27.6%)	0.07
**Median (IQR) ALT, U/L**	24.0 (18.0–34.0)	30.0 (21.0–44.0)	0.42
Missing	153 (2.6%)	341 (2.5%)	…
**Median (IQR) AST, U/L**	23.0 (19.0–30.0)	25.0 (20.0–34.0)	0.27
Missing	153 (2.6%)	341 (2.5%)	…
**Median (IQR) FIB-4 score**	1.1 (0.8–1.6)	1.1 (0.8–1.5)	0.05
**FIB-4 category**			0.09
<1.3	3442 (58.1%)	8291 (60.9%)	…
1.3–2.67	1912 (32.2%)	3910 (28.7%)	…
>2.67	288 (4.9%)	781 (5.7%)	…
Missing values	287 (4.8%)	635 (4.7%)	…
**Imaging modality**			0.50
Ultrasound	2050 (34.6%)	6324 (46.4%)	…
Computed tomography	2684 (45.3%)	6580 (48.3%)	…
Magnetic resonance	1168 (19.7%)	628 (4.6%)	…
Unspecified^d^	27 (0.5%)	85 (0.6%)	…

Abbreviations: ALT, alanine aminotransferase; AST, aspartate aminotransferase; AUD, alcohol use disorder; AUDIT-C, Alcohol Use Disorders Identification Test-Consumption; FIB-4, Fibrosis-4 Index for Liver Fibrosis; IQR, inter-quartile range; SLD, steatotic liver disease; Std Diff, standardized difference.

^a^Standardized difference of >0.10 is considered clinically significant.

^b^Other includes Asian, American Indian, or missing categorization.

^c^Alcohol use categories defined as follows: abstinent without AUD, AUDIT-C score of 0 and absence of AUD diagnosis; abstinent with AUD, AUDIT-C score of 0 and presence of AUD diagnosis; lower risk, AUDIT-C score of 1–3 and absence of AUD diagnosis; moderate risk, AUDIT-C score of 4–7 and absence of AUD diagnosis; high risk, AUDIT-C score of ≥8 or presence of AUD diagnosis with nonzero or missing AUDIT-C score.

^d^Report contained liver imaging results; however, we were unable to distinguish cross-sectional imaging modality as CT or MR.

### Incidence Rates and Cumulative Incidence of HCC/HD

Overall, 867 incident HCC/HD events (60 HCC; 807 HD) were identified more than 244 114 person-years of follow-up (composite incidence rate: 35.5 events/10 000 person-years). Median duration of follow-up was 12.5 years. The unadjusted incidence rate of HCC/HD was higher among people with SLD than without (41.3 vs 20.4 events/10 000 person-years; Supplementary Table 1). Unadjusted incidence rates of HCC/HD were similarly elevated among people ≥60 years old, people with diabetes, hypertension, and high-risk alcohol use. When results were stratified by SLD and HIV status, PWoH without SLD had the lowest rate of HCC/HD, PWH without SLD, and PWoH with SLD had higher incidence rates, whereas PWH with SLD had the highest incidence rate of these severe liver events (Figure 2).

**Figure 2.**
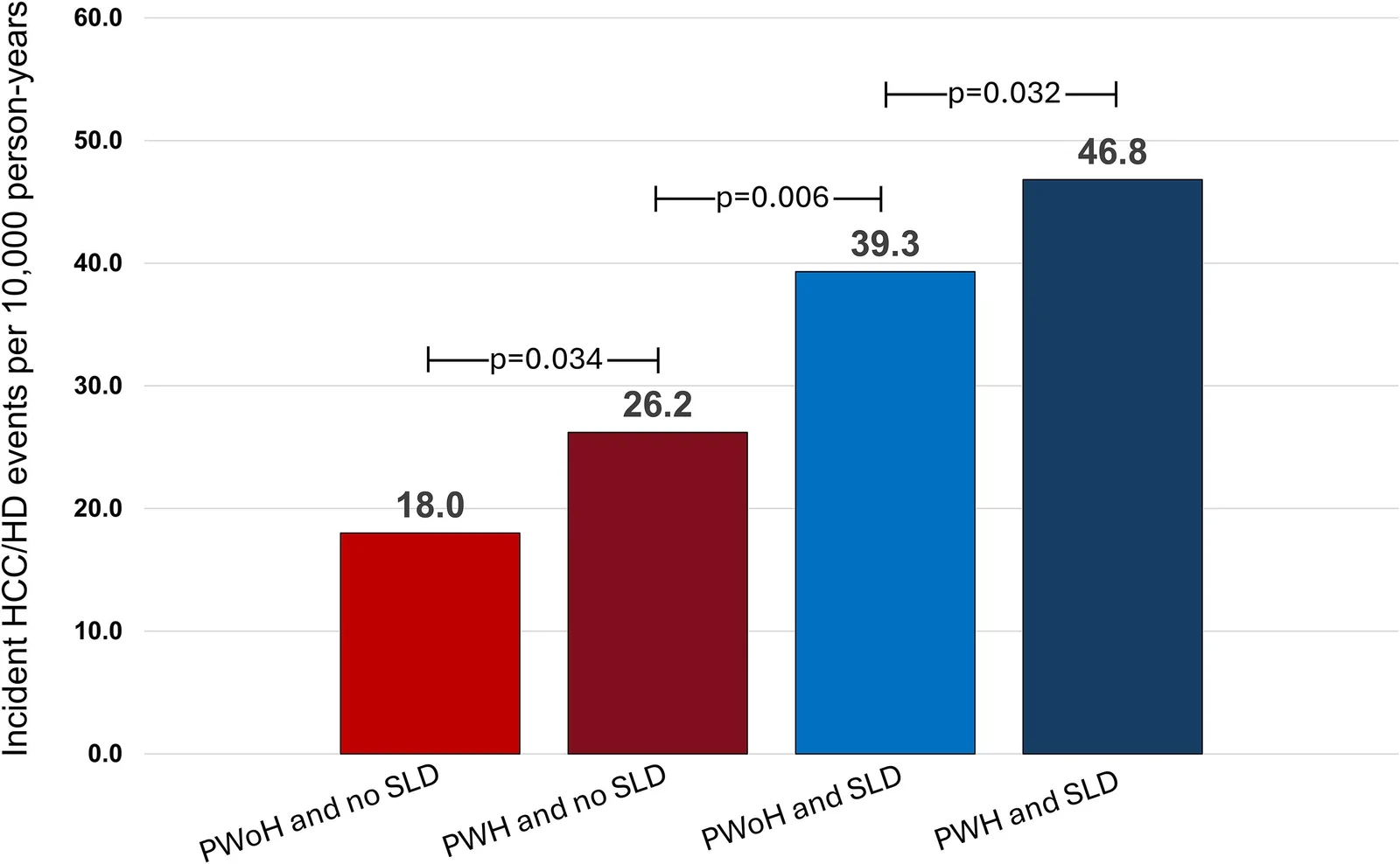
Incidence rates of composite outcome of hepatocellular carcinoma or hepatic decompensation (in events/10 000 person-y), by HIV and steatotic liver disease status. Abbreviations: HD, hepatic decompensation; PWH, people with HIV; PWoH, people without HIV; SLD, steatotic liver disease.

The cumulative incidence of HCC/HD in both PWoH and PWH differed by SLD status (Gray's test *P* < .0001; Figure 3). Among PWoH, the cumulative incidence of HCC/HD was 3.6% at 10 years in those with SLD, significantly greater when compared to 1.4% in those without SLD. Similarly, among PWH, the cumulative incidence of HCC/HD was 4.0% at 10 years in those with SLD, greater than the 2.6% cumulative incidence for PWH without SLD. After 15 years of follow-up, cumulative incidence of HCC/HD was highest among PWH and SLD at 6.3%; however, the cumulative incidence of HCC/HD among people with SLD did not differ significantly by HIV status (Supplementary Figure 1).

**Figure 3.**
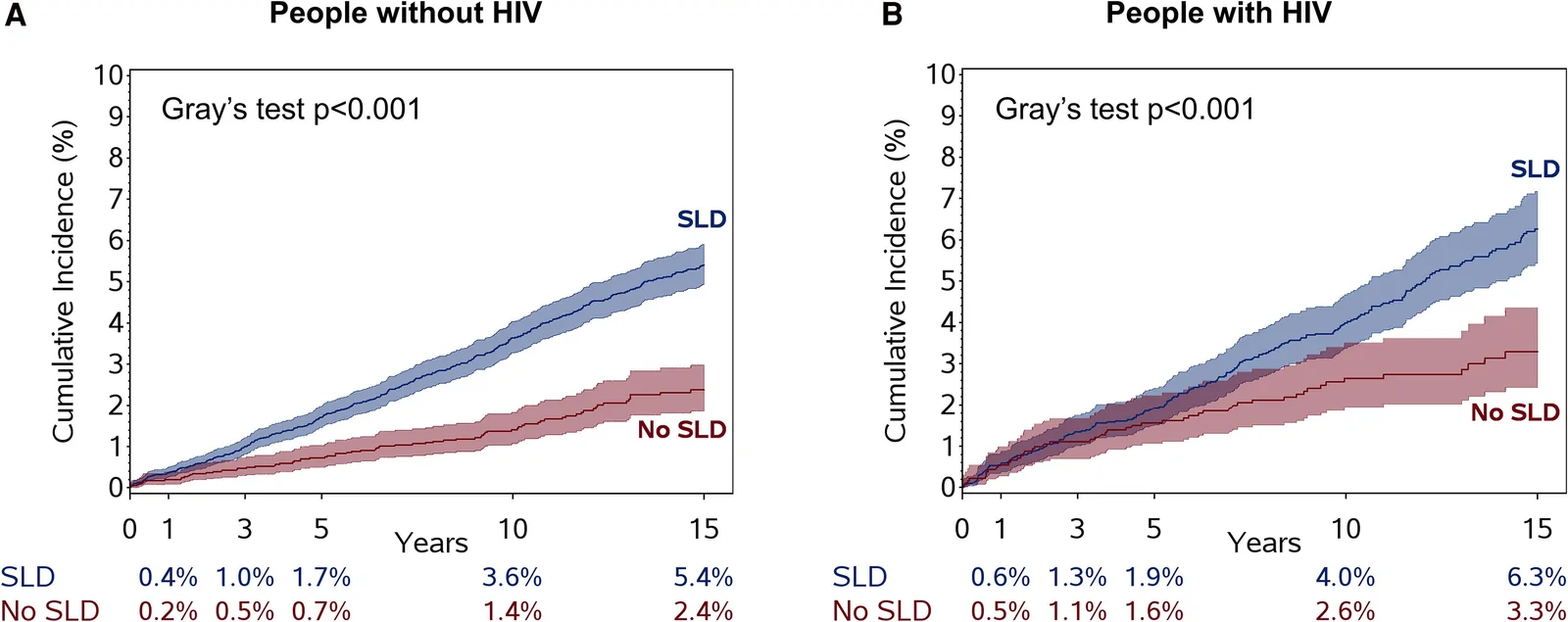
Cumulative incidence of composite outcome of hepatocellular carcinoma or hepatic decompensation in people with and without radiographic report of steatotic liver disease. (*A*) people without HIV; (*B*) people with HIV. Abbreviation: SLD, steatotic liver disease.

### Associations Between SLD and Incident HCC/HD

After adjustment for age, race, ethnicity, comorbidities, alcohol use category, and tobacco use, the risk of HCC/HD associated with SLD was highest among PWH and SLD (HR: 2.71; 95% CI, 2.10–3.49), followed by PWoH and SLD (HR: 2.09, 95% CI, 1.66–2.64) and PWH and no SLD (HR 1.62, 95% CI, 1.14–2.29) compared to PWoH and no SLD (Figure 4). No statistical interaction between HIV and SLD was observed (*P* value for interaction term: .25).

**Figure 4.**
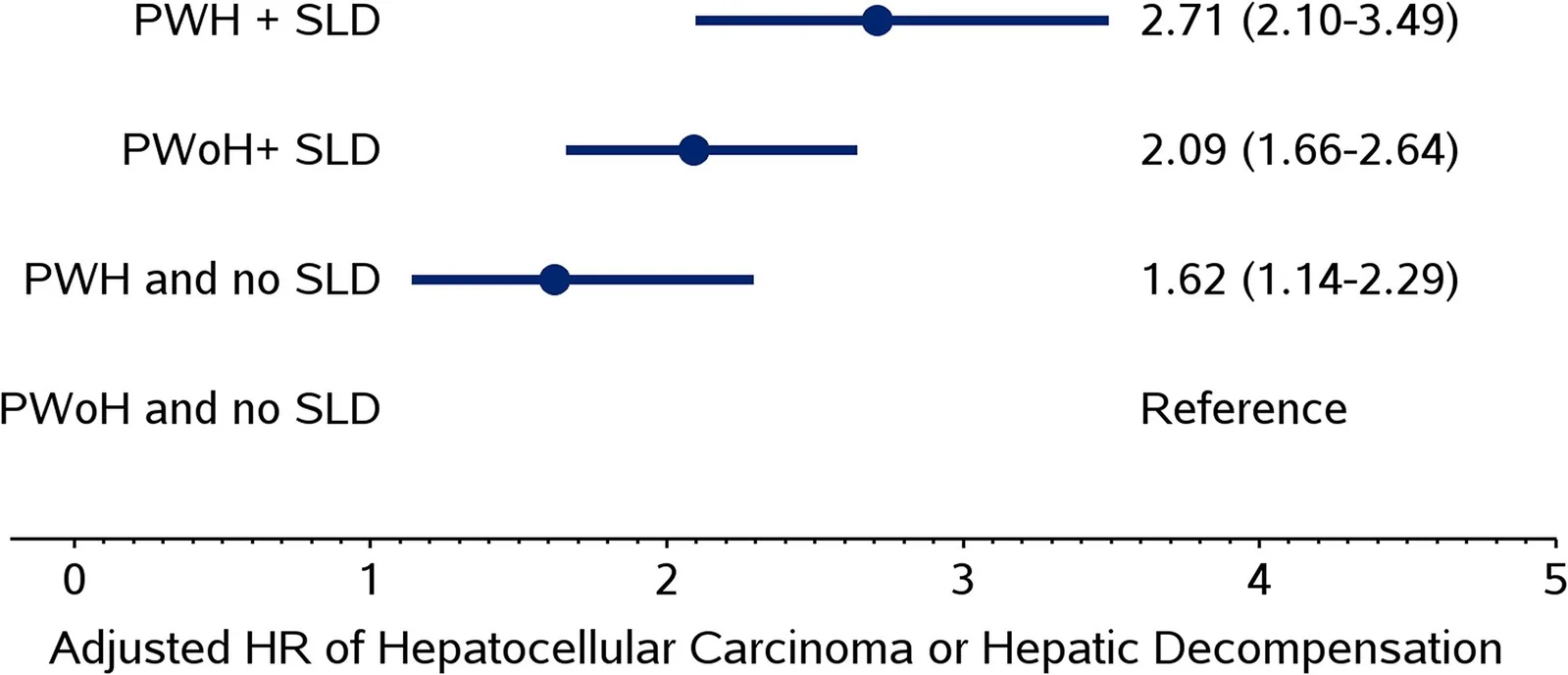
Adjusted hazards ratios and 95% confidence intervals of composite outcome of hepatocellular carcinoma or hepatic decompensation associated with steatotic liver disease and HIV status. Model additionally adjusted for age, sex, race/ethnicity, body mass index, tobacco, diabetes mellitus, hypertension, hyperlipidemia/statin use, and alcohol use category. Abbreviations: BMI, body mass index; PWH, people with HIV; PWoH, people without HIV; SLD, steatotic liver disease.

Other factors independently associated with greater risk of HCC/HD included increasing age, diabetes, hypertension, alcohol abstinence with alcohol use disorder diagnosis, or high-risk alcohol use when compared with lower-risk alcohol use (Table 2). Factors independently associated with lower risk of HCC/HD included non-Hispanic Black race, Hispanic ethnicity, and BMI of 30–34.9 kg/m^2^.

**Table 2.** Unadjusted and Adjusted Hazards Ratios of Hepatocellular Carcinoma or Hepatic Decompensation Associated With Groups Classified by Steatotic Liver Disease and HIV Status

Characteristics	Unadjusted HR (95% CI)	Adjusted HR^a^ (95% CI)
**Steatotic liver disease and HIV status**		
PWoH and no SLD	Ref	Ref
PWH and no SLD	**1.46** (**1.03–2.06)**	**1.62** (**1.14–2.29)**
PWoH + SLD	**2.17** (**1.72–2.72)**	**2.09** (**1.66–2.64)**
PWH + SLD	**2.58** (**2.01–3.32)**	**2.71** (**2.10–3.49)**
**Age, years**		
<40	Ref	Ref
40–49	**1.95** (**1.44–2.64)**	**1.68** (**1.24–2.28)**
50–59	**2.34** (**1.74–3.13)**	**1.89** (**1.40–2.55)**
≥60	**3.05** (**2.26–4.11)**	**2.51** (**1.84–3.44)**
**Female sex**	**0.56 (0.35–0.88)**	0.74 (0.47–1.16)
**Race/ethnicity**		
Non-Hispanic White	Ref	Ref
Non-Hispanic Black	**0.73** (**0.63–0.85)**	**0.71** (**0.61–0.82)**
Hispanic	**0.62** (**0.47–0.83)**	**0.59** (**0.44–0.79)**
Other^b^	0.92 (0.70–1.20)	0.87 (0.66–1.14)
Unknown	1.10 (0.75–1.63)	1.22 (0.83–1.80)
**Body mass index, kg/m^2^**		
<18.5	0.83 (0.39–1.77)	0.86 (0.40–1.82)
18.5–24.9	Ref	Ref
25.0–29.9	0.89 (0.74–1.08)	0.86 (0.71–1.05)
30.0–34.9	**0.79** (**0.64–0.97)**	**0.72** (**0.58–0.90)**
≥35.0	1.06 (0.86–1.31)	0.93 (0.74–1.17)
Missing	1.28 (0.87–1.88)	**1.51** (**1.02–2.24)**
**Tobacco use, ever**	**1.18** (**1.02–1.36)**	0.98 (0.84–1.13)
**Diabetes mellitus**	**1.87** (**1.62–2.16)**	**1.78** (**1.51–2.09)**
**Hypertension**	**1.56** (**1.35–1.79)**	**1.35** (**1.14–1.60)**
**Hyperlipidemia/statin use**		
Any statin use	1.13 (0.98–1.31)	0.90 (0.77–1.05)
Hyperlipidemia diagnosis, no statin use	0.94 (0.73–1.21)	1.13 (0.86–1.49)
No hyperlipidemia diagnosis, no statin use	Ref	Ref
**Alcohol use category** ^c^		
Abstinent without alcohol use disorder	1.09 (0.83–1.44)	1.00 (0.76–1.31)
Abstinent with alcohol use disorder	**2.04** (**1.44–2.88)**	**2.15** (**1.52–3.05)**
Lower risk	Ref	Ref
Moderate risk	1.14 (0.71–1.81)	1.16 (0.73–1.85)
High risk	**2.19** (**1.72–2.78)**	**2.30** (**1.80–2.95)**
Missing AUDIT-C	1.21 (0.96–1.53)	1.18 (0.94–1.49)

Statistically significant associations in bold. Abbreviations: AUD, alcohol use disorder; AUDIT-C, Alcohol Use Disorders Identification Test-Consumption; CI, confidence interval; HR, hazard ratio; PWH, people with HIV; PWoH, people without HIV; Ref, reference; SLD, steatotic liver disease.

^a^Adjusted for age, race, comorbidities, alcohol use category, and tobacco use.

^b^Other includes Asian, American Indian, or missing categorization.

^c^Alcohol use categories defined as follows: abstinent without AUD, AUDIT-C score of 0 and absence of AUD diagnosis; abstinent with AUD, AUDIT-C score of 0 and presence of AUD diagnosis); lower-risk, AUDIT-C score of 1–3 and absence of AUD diagnosis; moderate-risk, AUDIT-C score of 4–7 and absence of AUD diagnosis; high-risk, AUDIT-C score of ≥8 or presence of AUD diagnosis with nonzero or missing AUDIT-C score.

### Sensitivity Analyses

During the follow-up period, 3365 deaths were observed. Sensitivity analyses accounting for the competing risk of death demonstrated similar results as in the primary analysis (Supplementary Table 2). When limited to people with baseline FIB-4 < 2.67, associations were similar to those in the primary analysis (Supplementary Table 3). In exploratory analyses, unadjusted incidence rates of HCC/HD were highest among people with baseline FIB-4 > 2.67 across all groups (Supplementary Figure 2).

## DISCUSSION

Of the 19 546 patients without viral hepatitis or diagnosed cirrhosis included in our analyses, we found that radiographic report of SLD was highly prevalent in this sample of people who underwent liver imaging. Patients with SLD were more likely to be Hispanic, have a higher prevalence of cardiometabolic risk factors, higher risk alcohol use, but were not more likely to have HIV. When stratified by HIV and SLD status, PWH and SLD had the highest incidence rate of HCC/HD compared to PWoH and no SLD. People with SLD had >2-fold greater risk of HCC/HD, with PWH and SLD having the greatest risk of these severe liver events, after adjusting for demographics, cardiometabolic risk factors, alcohol, and tobacco use.

To our knowledge, this is the largest study to date evaluating rates of HCC/HD by HIV and SLD status, as defined by radiographic report of SLD. Prior work evaluating longitudinal liver-related outcomes of SLD by HIV status have been limited by smaller sample sizes, reducing the power to detect differences between groups. A recent study by Wong et al. found no difference in the incidence of cirrhosis or HCC by HIV status in a metabolic dysfunction-associated steatotic liver disease cohort, but the study had a small sample size (920 PWH) and relatively few events (169 decompensated cirrhosis and 39 HCC events) [49]. Moreover, people with chronic viral hepatitis were not excluded from the metabolic dysfunction-associated steatotic liver disease definition, preventing discrete evaluation of the association between SLD without viral hepatitis and liver complications. In contrast, our study included 3752 PWH and 9865 PWoH with radiologically defined SLD without chronic viral hepatitis or cirrhosis to evaluate incidence rates of hepatic decompensation or HCC, the most severe complications observed in the progression of chronic liver disease, demonstrating increased rates of these outcomes among PWH and SLD.

We observed a stepwise increase in risk of HCC/HD by HIV and SLD status, raising concern that SLD may be potentiated in the setting of HIV. Although we observed no statistically significant interaction between HIV and SLD, prior work has demonstrated increased risk of greater hepatic fibrosis development in PWH with SLD, suggesting accelerated hepatic fibrosis may occur in PWH and SLD [50]. Moreover, work comparing histopathologic severity of SLD demonstrated increased inflammation and fibrosis among PWH [8]. Additional research is needed to further understand factors associated with SLD progression in PWH to inform personalized treatment strategies.

Despite liver disease being a leading cause of death among PWH [6], and the high prevalence of SLD in PWH [51], there remains a paucity of data defining the risk of liver-specific complications associated with SLD in PWH or how these associations may differ from PWoH. Our work provides valuable insight into the increased risk of HCC/HD among those with radiographic findings of SLD in the absence of cardiometabolic risk factors or alcohol use. Closer clinical follow up may be indicated as radiographic finding of SLD may herald future development of cardiometabolic comorbidities. Because new pharmacologic interventions are approved for the treatment of SLD phenotypes in PWoH, it will be critical to understand how such therapies may impact PWH given the higher rates of HCC/HD in this group.

Our study has several potential limitations. First, we included the initial eligible imaging study to classify SLD status; as such, we could not capture the impact of interval development, differential resolution, or progression of SLD by HIV status during the follow-up period. Second, we adjusted our multivariable models for factors known to be associated with SLD; however, these variables were limited to baseline assessment thus changes in factors over time could not be assessed. Radiographic findings of SLD may precede diagnosis of metabolic comorbidities that may further increase the risk of HCC/HD, and these comorbidities may develop at different rates by HIV status. In contrast, behavioral or therapeutic interventions, such as weight loss or alcohol cessation, may ameliorate SLD and subsequently decrease the risk of HCC/HD. Our study did not account for such time-varying covariates; if our findings are replicated, future studies should evaluate the exacerbating or mitigating effects that changes in SLD may have on risk of HCC/HD. Third, our analyses are limited to people with imaging reports inclusive of the liver, thus may not be generalizable to people without liver imaging. However, the clinical identification of SLD is increasingly made by liver imaging [18, 42]. Our prior work demonstrated that people in VACS-HIV without eligible imaging studies are younger and with lower prevalence of cardiometabolic comorbidities [20]. Thus, the association between SLD and HCC/HD in this patient population without viral hepatitis is likely generalizable, particularly if these findings are confirmed in other cohorts. Fourth, the definition of SLD employed in these analyses required explicit notation within the radiographic reports. Although the NLP algorithm was previously validated within the VA and demonstrates excellent performance, there remains the potential for misclassification of exposure. Future work employing direct image analysis for identification of SLD is needed to overcome this potential for misclassification. Fifth, steatosis phenotypes, such as steatohepatitis or SLD with advanced fibrosis, were not examined in these analyses. Because steatohepatitis has historically required liver biopsy for diagnosis, and liver biopsy is rarely performed, such analyses would likely be underpowered for meaningful analysis.

Our study has several strengths. The VACS-HIV represents one of the largest observational cohorts of PWH and PWoH within care at the Veterans Health Administration, the largest integrated health system in the United States. Although VACS-HIV is predominantly male, the cohort includes >3000 women with HIV, representing one of the largest observational cohorts of women with HIV in the United States [52]. To ensure completeness of outcome ascertainment, we additionally linked to Medicare, decreasing the potential for under-ascertainment of HCC or HD events. We used validated definitions of HCC and HD to define our composite outcome, representing 2 forms of severe liver complications resulting from a common natural history of liver disease progression.

## CONCLUSIONS

Among people without cirrhosis or chronic viral hepatitis, radiographic findings of SLD are frequently observed and associated with increased risk of HCC/HD, independent of traditional risk factors including cardiometabolic factors and alcohol use. People with HIV and SLD were observed to have the highest rates of HCC/HD and greatest magnitude of risk for these severe liver events, though no statistical interaction between HIV and SLD was observed. Given the few HCC events observed, the risk of this event could not be evaluated separately. Future analyses should identify factors that may exacerbate or mitigate the progression of SLD to HCC in both PWH and PWoH, since such findings could inform personalized clinical risk stratification.

## Supplementary Material

ofag617_Supplementary_Data

## Supplementary Data

Supplementary materials are available at *Open Forum Infectious Diseases* online. Consisting of data provided by the authors to benefit the reader, the posted materials are not copyedited and are the sole responsibility of the authors, so questions or comments should be addressed to the corresponding author.
