# Supplementary material for: Risk of Hepatocellular Carcinoma or Hepatic Decompensation Associated With Steatotic Liver Disease in People With and Without HIV
Source: Open Forum Infect Dis. 2026 Oct 7;13(10):ofag617. doi: 10.1093/ofid/ofag617 (PMC13678219; doi:10.1093/ofid/ofag617)
Supplement: Supplementary file 1 [file ofag617_supplementary_data.docx]

**Risk of Hepatocellular Carcinoma or Hepatic Decompensation Associated with Steatotic Liver Disease in People with and without HIV**

Jessie Torgersen, MD, MHS, MSCE; Craig W. Newcomb, MS; Melissa Skanderson, MSW; Farah Kidwai-Khan, DEng; Dean M. Carbonari, MS; Janet P. Tate, MPH, ScD; Catherine Mezzacappa, MD, PhD; Emily J. Cartwright, MD; Debika Bhattacharya, MD; Joseph K. Lim, MD; Jennifer C. Price, MD, PhD; Tamar H. Taddei, MD; Amy C. Justice, MD, PhD; Vincent Lo Re III, MD, MSCE

**---SUPPLEMENTARY FILE---**

Supplementary Methods………………………………………………………………………2

Supplementary Table 1………………………………………………………………………..3

Supplementary Figure 1………………………………………………………………………5

Supplementary Table 2…..……………………………………………………………………6

Supplementary Table 3…..……………………………………………………………………8

Supplementary Figure 2…....……………………………………………….………………10

**Supplementary Methods**

The Alcohol Use Disorders Identification Test-Consumption (AUDIT-C) has been used as a universal screening test for harmful alcohol use in VA primary care since 2008. For the purposes of analysis, alcohol use was defined as a categorical variable including alcohol use disorder diagnoses coupled with the most recent AUDIT-C score. We defined alcohol use categories as follows: 1) abstinent without AUD (AUDIT-C=0 and absence of AUD diagnosis); 2) abstinent with AUD (AUDIT-C score=0 and presence of AUD diagnosis); 3) lower-risk alcohol use (AUDIT-C score=1-3 and absence of AUD diagnosis); 4) moderate-risk alcohol use (AUDIT-C score=4-7 and absence of AUD diagnosis); and 5) high-risk alcohol use (AUDIT-C score ≥8 or presence of AUD diagnosis with nonzero or missing AUDIT-C score).

**Supplementary Table 1: Unadjusted Incidence Rates of Composite Outcome of Hepatocellular Carcinoma or Hepatic Decompensation Among Patients with Clinical Liver Imaging in the VA Between October 1, 2001 and September 30, 2017 (n=19,546).**

| **Characteristics** | **Follow-up**  **(person-years)** | **Events** | **Incidence Rates per 10,000 person-years (95% CI)** |
| --- | --- | --- | --- |
| **Steatotic liver disease** |  |  |  |
| No | 67,217.22 | 137 | 20.4 (17.0-23.8) |
| Yes | 176,896.96 | 730 | 41.3 (38.3-44.3) |
| **Age, years** |  |  |  |
| <40 years | 31,998.72 | 52 | 16.3 (12.4-21.3) |
| 40-49 years | 70,284.15 | 224 | 31.9 (28.0-36.3) |
| 50-59 years | 88,189.20 | 333 | 37.8 (33.9-42.0) |
| ≥60 years | 53,642.12 | 258 | 48.1 (42.6-54.3) |
| **Sex** |  |  |  |
| Male | 234,728.09 | 848 | 36.1 (33.8-38.6) |
| Female | 9,386.10 | 19 | 20.2 (12.9-31.7) |
| **Race/ethnicity** |  |  |  |
| Non-Hispanic White | 105,791.60 | 437 | 41.3 (37.6-45.4) |
| Non-Hispanic Black | 96,289.30 | 291 | 30.2 (26.9-33.9) |
| Hispanic | 20,255.13 | 52 | 25.7 (19.6-33.7) |
| Other* | 15,815.22 | 60 | 37.9 (29.5-48.9) |
| Unknown | 5,962.94 | 27 | 45.3 (31.1-66.0) |
| **Body mass index, kg/m^2^** |  |  |  |
| <18.5 | 2,213.99 | 7 | 31.6 (15.1-66.3) |
| 18.5-24.9 | 46,766.20 | 179 | 38.3 (33.1-44.3) |
| 25.0-29.9 | 82,065.02 | 281 | 34.2 (30.5-38.5) |
| 30.0-34.9 | 63,872.81 | 194 | 30.4 (26.4-35.0) |
| ≥35.0 | 43,081.05 | 176 | 40.9 (35.2-47.4) |
| Missing | 6,115.12 | 30 | 49.1 (34.3-70.2) |
| **Tobacco use** |  |  |  |
| Never smoker | 84,002.49 | 267 | 31.8 (28.2-35.8) |
| Ever smoker | 160,111.69 | 600 | 37.5 (34.6-40.6) |
| **Diabetes** |  |  |  |
| No | 195,409.45 | 593 | 30.3 (28.0-32.9) |
| Yes | 48,704.73 | 274 | 56.3 (50.0-63.3) |
| **Hypertension** |  |  |  |
| No | 110,844.76 | 303 | 27.3 (24.4-30.6) |
| Yes | 133,269.43 | 564 | 42.3 (39.0-46.0) |
| **Hyperlipidemia/Statin Use** |  |  |  |
| Any statin use | 85,418.42 | 329 | 38.5 (34.6-42.9) |
| Hyperlipidemia diagnosis, no statin use | 21,490.46 | 69 | 32.1 (25.4-40.7) |
| No hyperlipidemia diagnosis, no statin use | 137,205.31 | 469 | 34.2 (31.2-37.4) |
| **Alcohol use category**^†^ |  |  |  |
| Abstinent without alcohol use disorder | 39,267.51 | 111 | 28.3 (23.5-34.0) |
| Abstinent with alcohol use disorder | 9,338.76 | 49 | 52.5 (39.7-69.4) |
| Lower-risk | 36,312.56 | 94 | 25.9 (21.1-31.7) |
| Moderate-risk | 7,473.39 | 22 | 29.4 (19.4-44.7) |
| High-risk | 40,498.03 | 232 | 57.3 (50.4-65.2) |
| Missing AUDIT-C | 111,223.93 | 359 | 32.3 (29.1-35.8) |
| **HIV infection** |  |  |  |
| No | 178,244.81 | 599 | 33.6 (30.9-36.3) |
| Yes | 65,869.37 | 268 | 40.7 (35.8-45.5) |
| Abbreviations: AUD, alcohol use disorder; AUDIT-C, Alcohol Use Disorders Identification Test-Consumption; CI, confidence interval; person-yrs, person-years  *Other includes Asian, American Indian, or missing categorization.  ^†^Alcohol use categories defined as follows: abstinent without AUD, AUDIT-C score of 0 and absence of AUD diagnosis; abstinent with AUD, AUDIT-C score of 0 and presence of AUD diagnosis); lower-risk, AUDIT-C score of 1-3 and absence of AUD diagnosis; moderate-risk, AUDIT-C score of 4-7 and absence of AUD diagnosis; high-risk, AUDIT-C score of ≥8 or presence of AUD diagnosis with nonzero or missing AUDIT-C score. | | | |

**Supplementary Figure 1. Cumulative Incidence of Composite Outcome of Hepatocellular Carcinoma or Hepatic Decompensation by HIV and Steatotic Liver Disease Status.**

**Supplementary Table 2: Unadjusted and Adjusted Hazards Ratios of Hepatocellular Carcinoma or Hepatic Decompensation in Patients with Clinical Liver Imaging Reports Without Baseline Cirrhosis or Chronic Viral Hepatitis, Accounting for Competing Risk of Death.**

| **Characteristics** | **Unadjusted HR**  **(95% CI)** | **Adjusted HR***  **(95% CI)** |
| --- | --- | --- |
| **Steatotic liver disease and HIV status** |  |  |
| PWoH and no SLD | Ref | Ref |
| PWH and no SLD | **1.40 (0.99-1.98)** | **1.52 (1.07-2.16)** |
| PWoH + SLD | **2.25 (1.79-2.83)** | **2.14 (1.70-2.70)** |
| PWH +SLD | **2.59 (2.02-3.32)** | **2.65 (2.06-3.40)** |
| **Age, years** |  |  |
| <40 years | Ref | Ref |
| 40-49 years | **1.92 (1.42-2.60)** | **1.67 (1.23-2.27)** |
| 50-59 years | **2.16 (1.61-2.90)** | **1.77 (1.31-2.40)** |
| ≥60 years | **2.44 (1.81-3.29)** | **2.07 (1.51-2.85)** |
| **Female sex** | **0.60 (0.38-0.94)** | 0.75 (0.48-1.19) |
| **Race/ethnicity** |  |  |
| Non-Hispanic White | Ref | Ref |
| Non-Hispanic Black | **0.73 (0.63-0.85)** | **0.71 (0.61-0.82)** |
| Hispanic | **0.63 (0.47-0.84)** | **0.60 (0.45-0.80)** |
| Other^†^ | 0.93 (0.71-1.22) | 0.88 (0.67-1.16) |
| Unknown | 1.02 (0.69-1.51) | 1.12 (0.76-1.66) |
| **Body mass index, kg/m^2^** |  |  |
| <18.5 | 0.66 (0.31-1.42) | 0.67 (0.31-1.45) |
| 18.5-24.9 | Ref | Ref |
| 25.0-29.9 | 0.94 (0.78-1.14) | 0.92 (0.76-1.11) |
| 30.0-34.9 | 0.85 (0.69-1.04) | **0.78 (0.63-0.97)** |
| ≥35.0 | 1.13 (0.92-1.39) | 0.99 (0.78-1.24) |
| Missing | 1.32 (0.89-1.95) | 1.48 (0.99-2.22) |
| **Tobacco use, ever** | 1.13 (0.98-1.31) | 0.94 (0.81-1.09) |
| **Diabetes** | **1.72 (1.49-1.98)** | **1.69 (1.43-2.00)** |
| **Hypertension** | **1.46 (1.27-1.68)** | **1.32 (1.11-1.57)** |
| **Hyperlipidemia/Statin Use** |  |  |
| Any statin use | 1.09 (0.95-1.25) | 0.89 (0.76-1.05) |
| Hyperlipidemia diagnosis, no statin use | 0.98 (0.76-1.26) | 1.16 (0.88-1.53) |
| No hyperlipidemia diagnosis, no statin use | Ref | Ref |
| **Alcohol use category**^‡^ |  |  |
| Abstinent without alcohol use disorder | 1.09 (0.83-1.44) | 0.96 (0.73-1.26) |
| Abstinent with alcohol use disorder | **2.04 (1.44-2.88)** | **1.96 (1.38-2.78)** |
| Lower-risk | Ref | Ref |
| Moderate-risk | 1.14 (0.71-1.81) | 1.18 (0.74-1.89) |
| High-risk | **2.19 (1.72-2.78)** | **2.28 (1.78-2.92)** |
| Missing AUDIT-C | 1.21 (0.96-1.53) | 1.23 (0.97-1.54) |
| Abbreviations: AUD, alcohol use disorder; AUDIT-C, Alcohol Use Disorders Identification Test-Consumption; CI, confidence interval; HR, hazard ratio; PWH, people with HIV; PWoH, people without HIV; Ref, reference; SLD, steatotic liver disease  *Adjusted for age, race, comorbidities, alcohol use category, and tobacco use.  ^†^Other includes Asian, American Indian, or missing categorization.  ^‡^Alcohol use categories defined as follows: abstinent without AUD, AUDIT-C score of 0 and absence of AUD diagnosis; abstinent with AUD, AUDIT-C score of 0 and presence of AUD diagnosis); lower-risk, AUDIT-C score of 1-3 and absence of AUD diagnosis; moderate-risk, AUDIT-C score of 4-7 and absence of AUD diagnosis; high-risk, AUDIT-C score of ≥8 or presence of AUD diagnosis with nonzero or missing AUDIT-C score. | | |

**Supplementary Table 3: Unadjusted and Adjusted Hazards Ratios of Hepatocellular Carcinoma or Hepatic Decompensation Associated with Groups Classified by Steatotic Liver Disease and HIV Status, After Additionally Excluding Patients with Baseline FIB-4 >2.67 (n=18,472).**

| **Characteristics** | **Unadjusted HR**  **(95% CI)** | **Adjusted HR***  **(95% CI)** |
| --- | --- | --- |
| **Steatotic liver disease and HIV status** |  |  |
| PWoH and no SLD | Ref | Ref |
| PWH and no SLD | **1.30 (0.89-1.92)** | **1.52 (1.03-2.24)** |
| PWoH + SLD | **1.91 (1.49-2.44)** | **1.84 (1.44-2.36)** |
| PWH +SLD | **2.35 (1.80-3.09)** | **2.58 (1.96-3.41)** |
| **Age, years** |  |  |
| <40 years | Ref | Ref |
| 40-49 years | **1.85 (1.34-2.55)** | **1.59 (1.15-2.20)** |
| 50-59 years | **2.16 (1.58-2.96)** | **1.70 (1.23-2.35)** |
| ≥60 years | **2.85 (2.06-3.94)** | **2.22 (1.58-3.11)** |
| **Female sex** | **0.57 (0.35-0.94)** | 0.73 (0.45-1.21) |
| **Race/ethnicity** |  |  |
| Non-Hispanic White | Ref | Ref |
| Non-Hispanic Black | **0.74 (0.62-0.87)** | **0.72 (0.61-0.85)** |
| Hispanic | **0.58 (0.42-0.81)** | **0.56 (0.40-0.79)** |
| Other^†^ | 0.99 (0.74-1.33) | 0.95 (0.70-1.27) |
| Unknown | 1.00 (0.63-1.59) | 1.10 (0.69-1.74) |
| **Body mass index, kg/m^2^** |  |  |
| <18.5 | 0.88 (0.36-2.15) | 0.90 (0.37-2.21) |
| 18.5-24.9 | Ref | Ref |
| 25.0-29.9 | 1.01 (0.82-1.26) | 0.96 (0.77-1.20) |
| 30.0-34.9 | 0.87 (0.69-1.10) | 0.79 (0.61-1.00) |
| ≥35.0 | 1.16 (0.92-1.48) | 0.99 (0.76-1.29) |
| Missing | 1.23 (0.77-1.95) | 1.46 (0.91-2.33) |
| **Tobacco use, ever** | **1.24 (1.05-1.46)** | 1.05 (0.89-1.25) |
| **Diabetes** | **1.90 (1.62-2.23)** | **1.73 (1.44-2.07)** |
| **Hypertension** | **1.62 (1.39-1.90)** | **1.36 (1.13-1.65)** |
| **Hyperlipidemia/Statin Use** |  |  |
| Any statin use | **1.24 (1.06-1.46)** | 0.99 (0.83-1.18) |
| Hyperlipidemia diagnosis, no statin use | 0.84 (0.62-1.14) | 0.99 (0.72-1.37) |
| No hyperlipidemia diagnosis, no statin use | Ref | Ref |
| **Alcohol use category**^‡^ |  |  |
| Abstinent without alcohol use disorder | 1.26 (0.92-1.72) | 1.15 (0.84-1.57) |
| Abstinent with alcohol use disorder | **2.29 (1.55-3.39)** | **2.35 (1.58-3.48)** |
| Lower-risk | Ref | Ref |
| Moderate-risk | 1.24 (0.73-2.11) | 1.25 (0.74-2.13) |
| High-risk | **2.09 (1.57-2.78)** | **2.17 (1.62-2.91)** |
| Missing AUDIT-C | **1.34 (1.03-1.76)** | 1.31 (1.00-1.72) |
| Abbreviations: AUD, alcohol use disorder AUDIT-C, Alcohol Use Disorders Identification Test-Consumption; CI, confidence interval; HR, hazard ratio; PWH, people with HIV; PWoH, people without HIV; Ref, reference; SLD, steatotic liver disease  *Adjusted for age, race, comorbidities, alcohol use category, and tobacco use.  ^†^Other includes Asian, American Indian, or missing categorization.  ^‡^Alcohol use categories defined as follows: abstinent without AUD, AUDIT-C score of 0 and absence of AUD diagnosis; abstinent with AUD, AUDIT-C score of 0 and presence of AUD diagnosis); lower-risk, AUDIT-C score of 1-3 and absence of AUD diagnosis; moderate-risk, AUDIT-C score of 4-7 and absence of AUD diagnosis; high-risk, AUDIT-C score of ≥8 or presence of AUD diagnosis with nonzero or missing AUDIT-C score. | | |

**Supplementary Figure 2. Incidence Rates of Hepatocellular Carcinoma or Hepatic Decompensation (in events/10,000 person-years) with 95% Confidence Intervals by HIV, Steatotic Liver Disease Status, and FIB-4 Category.**

Abbreviations: FIB-4, Fibrosis-4 Index; HCC, hepatocellular carcinoma; HD, hepatic decompensation; PWH, people with HIV; PWoH, people without HIV; SLD, steatotic liver disease
